# Can Infrared Spectroscopy Be Used to Measure Change in Potassium Nitrate Concentration as a Proxy for Soil Particle Movement?

**DOI:** 10.3390/s110404188

**Published:** 2011-04-07

**Authors:** Mila Ivanova Luleva, Harald van der Werff, Victor Jetten, Freek van der Meer

**Affiliations:** Faculty of Geo-Information Science and Earth Observation (ITC), University of Twente, Hengelosestraat 99, P.O. Box 37, 7500AA, Enschede, The Netherlands; E-Mails: vdwerff@itc.nl (H.W.); jetten@itc.nl (V.J.); vdmeer@itc.nl (F.M.)

**Keywords:** soil particles, soil erosion, chemical tracer, Potassium, infrared spectroscopy, absorption feature parameters

## Abstract

Displacement of soil particles caused by erosion influences soil condition and fertility. To date, the cesium 137 isotope (^137^Cs) technique is most commonly used for soil particle tracing. However when large areas are considered, the expensive soil sampling and analysis present an obstacle. Infrared spectral measurements would provide a solution, however the small concentrations of the isotope do not influence the spectral signal sufficiently. Potassium (K) has similar electrical, chemical and physical properties as Cs. Our hypothesis is that it can be used as possible replacement in soil particle tracing. Soils differing in texture were sampled for the study. Laboratory soil chemical analyses and spectral sensitivity analyses were carried out to identify the wavelength range related to K concentration. Different concentrations of K fertilizer were added to soils with varying texture properties in order to establish spectral characteristics of the absorption feature associated with the element. Changes in position of absorption feature center were observed at wavelengths between 2,450 and 2,470 nm, depending on the amount of fertilizer applied. Other absorption feature parameters (absorption band depth, width and area) were also found to change with K concentration with coefficient of determination between 0.85 and 0.99. Tracing soil particles using K fertilizer and infrared spectral response is considered suitable for soils with sandy and sandy silt texture. It is a new approach that can potentially grow to a technique for rapid monitoring of soil particle movement over large areas.

## 1. Introduction

Land degradation is a relatively slow process [1]. Physical and chemical degradation, under the influence of wind and water, leads to loss of nutrients, soil instability, subsoil exposure and desertification. Well-known erosion features such as rills and gullies are manifestations of an already advanced degradation [2,3]. To detect early warning signs, however, it is important to monitor soil properties sensitive to degradation, such as chemical composition, runoff and sediment yield. Natural variation in soil chemical composition is associated with bedrock geology and soil type, although agricultural practices and overgrazing also influence surface soil chemistry and quality [4–6]. Hence, studies on soil erosion have focused on using soil chemical composition mainly for particle tracing.

Various chemical soil particle tracers have been used to obtain spatially distributed data for soil erosion [7] and used to identify suspended sediment [8]. Commonly used soil particle tracers are the cesium 137 isotope (^137^Cs) [9–15], lead (^210^Pb) and beryllium (^7^Be) [16,17], and rare earth oxides [7,18]. Although ^137^Cs is considered the primary chemical tracer for detection of soil particle movement [19–23], one has to assume a homogeneous distribution of ^137^Cs fall out limited to the Northern hemisphere, and that all particle movements are a result of soil erosion [13,24,25]. Cost of soil sampling and analysis and the limited half-life of the element are the main limitations to extrapolate these methods to cover large areas [2].

Soil properties have been studied with infrared spectroscopy since the 80's, using visible, near-infrared and shortwave infrared wavelength region (400–2,500 nm). Spectral reflectance is determined by both physical and chemical characteristics of soils [26–28]. Soil spectral features are mainly a result of overtone absorption and combination of bond vibrations in molecules of three functional groups in minerals: OH, SO_4_ and CO_3_ [29,30]. Organic matter is also found to have influence on spectral response since it holds most positively charged nutrients in soils. However, due to the relatively weak attraction between K and this soil constituent, K absorption is not found to be affected [31]. Results obtained using regression models for detection of soluble fractions of potassium, only have moderate accuracy and vary according to study sites [32]. Sampling large areas for determination of soil properties using spectral reflectance is relatively cheap and fast, compared to traditional field and laboratory techniques [28]. To date, infrared spectra have not been put in use when studying soil erosion with ^137^Cs. Low concentrations of the isotope in nature makes the identification of the element through spectral means impossible, considering the capabilities of available spectrometers [33].

The element potassium (K) shares electrical, chemical and physical properties with Cs, both being members of the Group I alkali metals [34,35]. Both elements have similar biological and chemical behaviour, where the difference is only in reactivity [34], but it has not been tested as a particle tracer. Potassium occurs naturally in the environment, but it is also used on agricultural lands as a fertilizer. The amount of K fertilizer (in a form of K_2_O or K-P-N) typically applied by farmers, according to EU Directives: Nitrates Directive (91/676/EC) and Water Framework Directive (2000/60/EC), as well as EU commission recommendations, is 183 kg of solid fertilizer per hectare or in dissolved solution 1.83 g of solid fertilizer per 10 mL of water for sandy silt soils, although there is no specific policy adopted for the EU region. In practice, the range of totals of applied amount may reach 5–8 g/10 mL according to soil type and water conditions. In soils, K is mainly present as part of preliminary soil minerals (unavailable), in clay minerals and fine silt (slowly available), and in a water-soluble form (readily available) [36–38]. When K fertilizers are applied, they dissolve and K becomes part of the soil-water solution [38]. Fertilizers are applied prior to harvesting. To maintain optimal crop production, uniform application of the fertilizer is the most desired practice [39]. Potassium remains immobile as long as soils are not chopped or plowed. The mobility and distribution of this element has also been discussed by [40–42]. These authors explain the behaviour of the element determined by soil physical properties such as texture, porosity, organic matter and water holding capacity. They also state that due to low cation exchange capacity and organic matter content, sandy soils are more likely to experience K leaching than clays. Furthermore, soil texture combined with chemical composition (presence of calcium, sodium or gypsum, of both soil and water used for irrigation) determine the rates of leaching of K [41,43]. Establishing the behaviour of K in soils can prevent under or overestimating of predicted sediment deposits in studies where the element is used as a tracer.

The aim of this paper was to determine the spectral characteristics of K in soils and its capability to replace ^137^Cs in large scale soil erosion monitoring. This is examined by measuring infrared spectral response of soils of different texture. The laboratory analysis comprises of two experimental stages, with varying concentrations of added K fertilizer. Fertilizer concentrations, in range of three times higher than typically applied by farmers, are used to quantify K through spectral analysis and establish wavelength ranges sensitive to change in concentration of the element. The findings are subsequently compared to literature based on Partial Least Squares Regression modelling and derivative manipulations. The second stage of the experiment includes application of typically used in agricultural field concentrations, to determine the lowest detectable concentrations.

## 2. Methods

### 2.1. Laboratory Experiments

Eight locations in central, east and southeast Netherlands were chosen for soil sample collection, according to soil type and soil texture. The collected material varied from heavy clay (Fluvisol-Eutric), clay loam (Fluvaquent-Typic), loam (Fluvisol-Calcaric), silty loam (Luvisol-Orthic) and sandy loam (coarse sand) (Podzol-Humic), to fine sand (Arenosols-Albic). Description of each soil type can be found in Table 1.

At each location, 1–2 kg of material was collected using a clean spade to form a composite sample of the surface soil (0–5 cm). Each composite sample comprised of ten combined discrete samples collected around a single location, in order to represent all components of the sampled body material. Duplicates were acquired for silty and sandy loam soils. The samples were air dried for two days and subsequently sieved to remove particles larger than 5 mm.

First, each sample was divided and placed in four identical metal dishes (30 g of soil each). The soil material in three of the dishes was fertilized with respectively 8, 16 and 32 g/10 mL solution of potassium nitrate fertilizer (46% K_2_O 13% N, organic matter). The use of fertilizer in the experiment allowed strict control over the added concentrations. To the fourth, demineralised water was added for use as a reference. Prior to acquiring spectra, samples were dried overnight at 30 °C, to remove the effect of moisture on the spectral reflectance. The percentage Sand-Silt-Clay content was determined through bulk density and soil texture analysis, following standard laboratory procedures [45]. An Analytical Spectral Devices (ASD) Fieldspec Pro Spectrometer with a 450–2,500 nm wavelength range coverage at 2–3 nm spectral resolution was used to acquire reflectance spectra. A high-intensity contact probe with internal light source was used as fore-optic with a spot size of 10 mm. An average spectrum was created per soil textural type and amount of K, based on 10 measurements repeated for 20 iterations.

In the second stage of the experiment, three soil textural types (heavy clay, fine sand and silt loam) were selected, as these clearly show the effect of each textural component (sand, silt, clay) on spectra. The same procedure as above was followed, but now nine dilutions of fertilizer in the range of 1.13 g/10 mL to 1.93 g/10 mL were applied to each soil class, and one set was used as a reference. Spectra were collected in the same setup as described above.

### 2.2. Absorption Feature Characteristics

**Figure 1.**
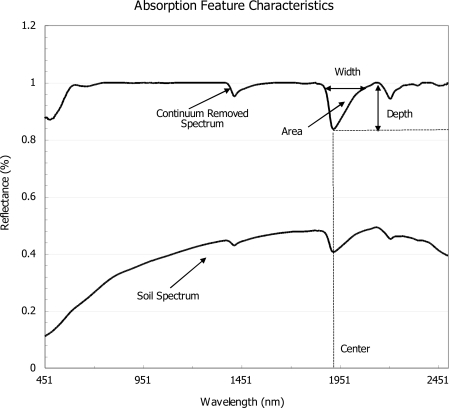
Original spectra and continuum removed spectra showing absorption feature parameters: absorption-band depth, width, center and area.

To determine the wavelength range influenced by change in K concentrations, and to compare the parameters from a common base line, an absorption feature analysis using continuum removal was performed [46]. This method is found to enhance differences in shape between individual features [47]. Mathematically, it is calculated by dividing each individual spectrum by the corresponding continuum line [48]. Absorption features were associated with K through sensitivity analysis to assess the impact of added fertilizer on specific absorption features. Coefficients of determination were calculated based on changes in absorption caused by ranging concentrations of added fertilizer. After identifying the wavelength range related to K, absorption feature characteristics including absorption depth, absorption center, absorption area and absorption width (Figure 1) of the individual feature were calculated using IDL-ENVI software [49]. Equations implemented in IDL scripts are reported in [50]. Coefficients of determination were calculated to identify trends associated with characteristics of absorption features in regards to changes in concentration levels and soil textural type.

## 3. Results

### 3.1. Laboratory Testing of Potassium Influence on Spectra

**Figure 2.**
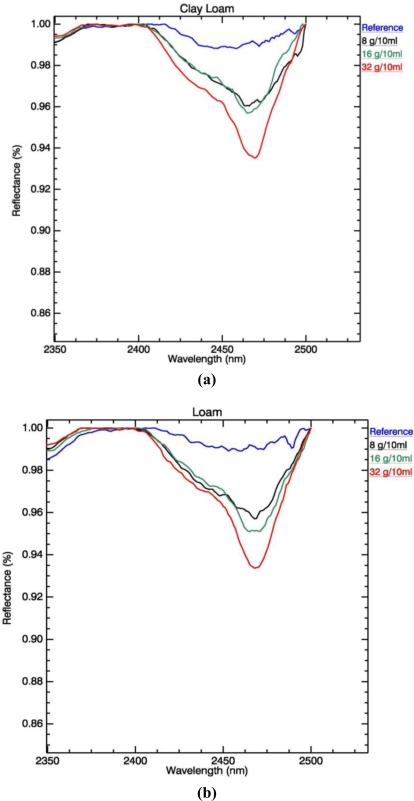

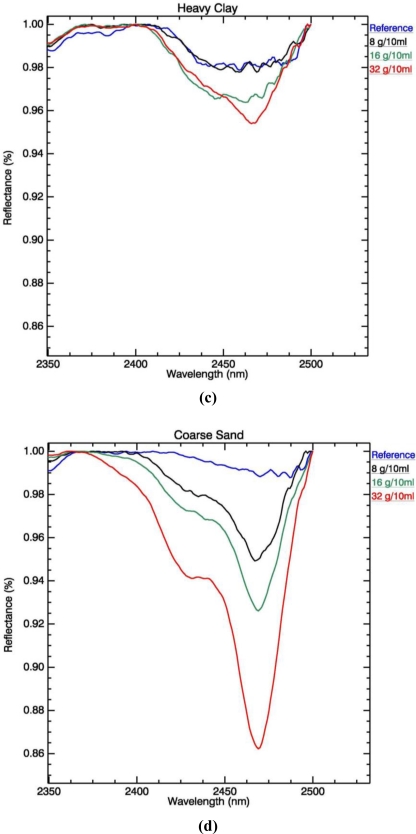

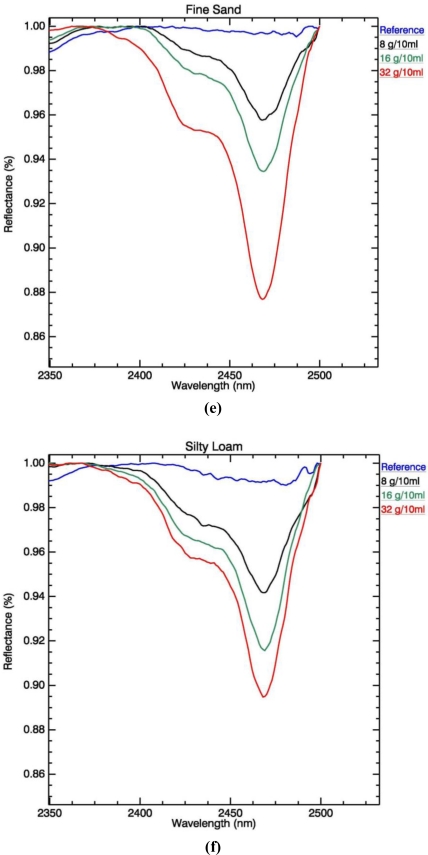
Change in absorption at 2,450–2,470 nm with change in K concentration. Each spectrum is an average of 10 measurements. The blue lines belong to soils with no added fertilizer and used as a reference. The deeper absorption features belong to samples with addition of K fertilizer in concentrations 8, 16 and 32 g/10 mL, indicated with black, green and red line respectively. The sub-figures show: **(a)** Clay Loam, **(b**) Loam, **(c)** Heavy clay; **(d)** Coarse sand, **(e)** Fine sand, **(f)** Silty loam.

**Figure 3.**
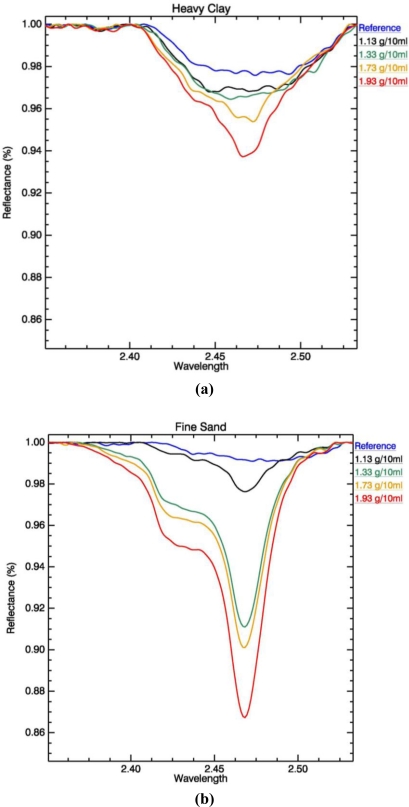

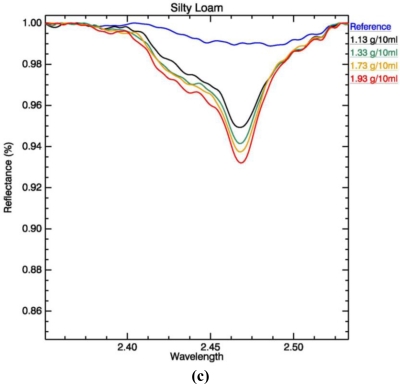
Change in absorption at 2,450–2,470 nm with change in K concentration. Each spectrum is an average of 10 measurements. The blue lines belong to soils with no added fertilizer and used as a reference. The deeper absorption features belong to samples with addition of K fertilizer in concentrations 1.13 g/10 mL (black), 1.33 g/10 mL (green), 1.73 g/10 mL (orange) and 1.93 g/10 mL (red); **(a)** Heavy clay, **(b)** Fine Sand, **(c)** Silty Loam.

Based on results from continuum removal and sensitivity analyses, the wavelength range that relates to potassium concentration was found to be 2,450–2,470 nm. This is based on the highest estimated coefficients of determination (R^2^) for change in absorption in regards to K concentration. For this wavelength range, values vary between 0.918 for clay loam samples to 0.984 for silty loam samples. Figure 2 shows changes in absorption at 2,450–2,470 nm caused by changes in K concentration, based on the first stage of laboratory experiments. The deepest point of absorption was observed between 2,450 to 2,470 nm. With addition of fertilizer, the depth of absorption increases accordingly. This is also observed when small quantities of fertilizer were applied (Figure 3). This figure shows changes in absorption after application of fertilizer in quantities between 1.13 g/10 mL and 1.93 g/10 mL, for the heavy clays, fine sand and silt loam soil samples. Changes are noted for fine sand and silt loam samples, the absorption in the heavy clay soil samples is not clear.

### 3.2. Absorption Feature Analysis

**Figure 4.**
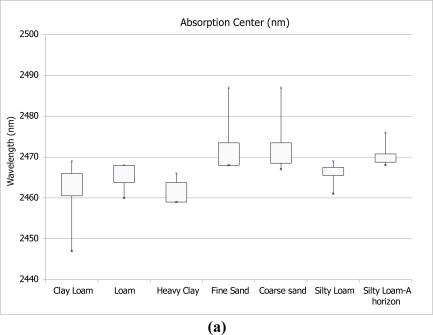

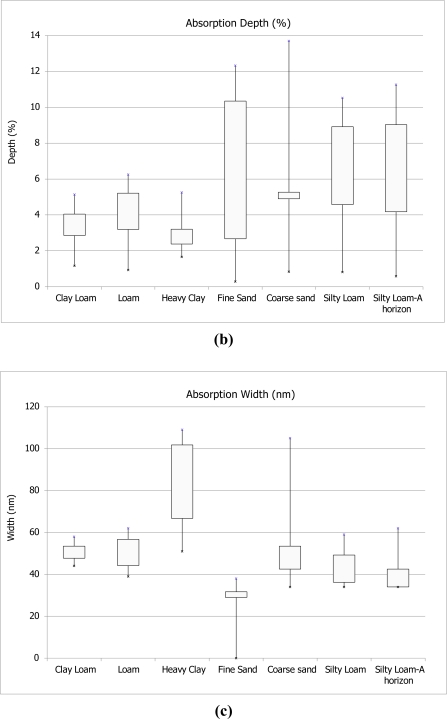
Variation in absorption feature parameters per soil textural type. Texture classes in dark grey contain highest clay content, lighter grey-highest sand content and the lightest grey-highest silt content: (**a)** Absorption center, (**b)** Absorption depth, (**c)** Absorption width.

Figure 4 shows variation per soil textural type in absorption depth, width and center of absorption in the 2,450–2,470 nm wavelength range.

**Figure 5.**
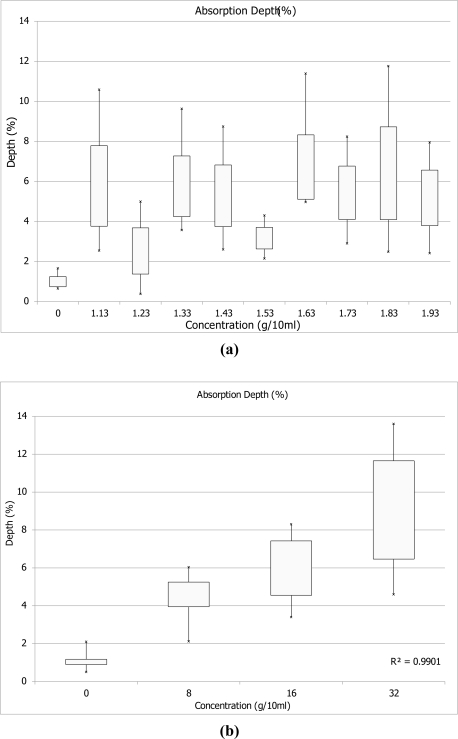

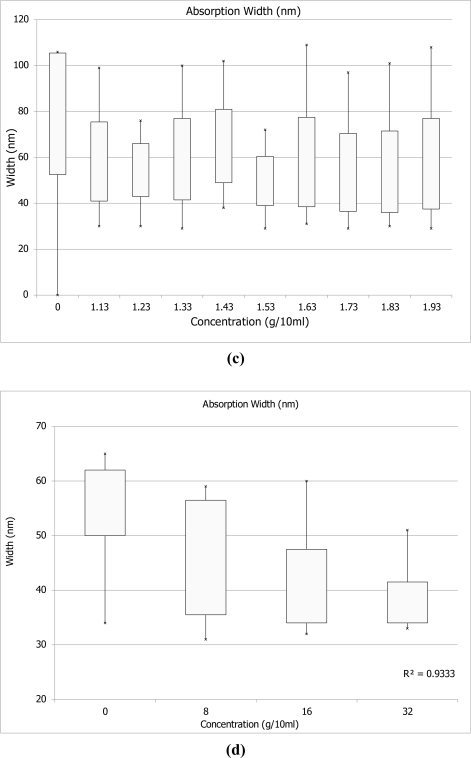

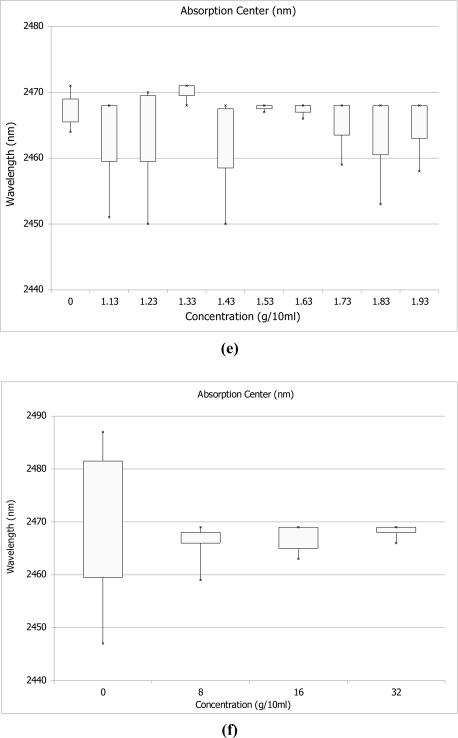
Change in absorption feature parameters-depth of the absorption, width and center, classes based on varying concentrations of added K fertilizer 0 to 32 g/10 mL for all samples and soil textural types: **(a)** Absorption band depth for 0 to 1.93 g/10 mL, **(b)** Absorption band depth for 0 to 32 g/10 mL, **(c)** Absorption width for 0 to 1.93 g/10 mL, **(d)** Absorption width for 0 to 32 g/10 mL, **(e)** Absorption center for 0 to 1.93 g/10 mL, **(f)** Absorption center for 0 to 32 g/10 mL.

Figure 5 shows changes in absorption feature characteristics due to increase in K concentrations. Both figures show that the spectral response of soils with higher clay content is characterised by lower R^2^ values and noisier spectrum. A shift of 5–10 nm in center of absorption is most profound in the clay samples, although all samples show a shift to lower wavelengths with increase of K concentration. An increase in band depth is noted for soil samples that belong to the silty loam textural class. This cannot be observed for the heavy clay and fine sand samples.

## 4. Discussion

### 4.1. Potassium Influence on Spectra

From continuum removal and sensitivity analysis performed on laboratory derived spectra, the wavelength range where the center of K absorption can be found is 2,450 to 2,470 nm. This falls within the range reported in literature using second derivative calculations [30] and Partial Least Squares Regression models [51]. These studies reported optimal calibration equations for K that produce R^2^ of 0.89 for wavelengths between 2,396 and 2,462 nm. The characteristic feature can be accounted to possible absorptions of combination modes between the free K ions and OH functional group.

Minor change in depth of absorption with addition of K fertilizer, is observed in samples with high clay content, that belong to soil textural classes clay loam and heavy clay [Figures 2(a–c) and 3(a)]. This is explained by the fact that clay content in samples prevents significant spectral changes caused by non-clay minerals [27]. Potassium is held at the edges of the clay particles, causing easy replacement by other positive ions [52]. The soil textural types that show the highest R^2^ between K concentration and depth of absorption are also the ones most prone to soil erosion: Silty loam and fine sand [Figures 2(d–f) and 3(b,c)].

### 4.2. Absorption Feature Analysis

The center wavelength and depth of the K absorption feature becomes better defined with an increase in concentration of added fertilizer. This is observed in the findings that emerge from both stages of laboratory analysis for all soil textural types (Figures 2 and 3). Changes in absorption feature parameters appear when 8, 16 and 32 g/10 mL of fertilizer are applied. Figure 4(a) shows that soil texture defines the position of K absorption center. High clay content in soils causes greater fluctuations in absorption center values. The increase in concentration, as seen in Figure 5(e,f), has strong influence on the peak position only above 8 g/10 mL of fertilizer. The effect K concentration has on spectra is highlighted in the case of absorption band depth with R^2^ value of 0.99 for concentrations higher than 8 g/10 mL [Figure 5(b)].

Samples with high clay content experience smaller changes in absorption, and have lower coefficients of determination compared to those belonging to highly erosive soil textural types-sand and silt loam. This is explained by the abundance of free K ions in clays and the formation of illite-like [53] groups, where K replaces water and fills the interlayer clay sites. The presence of clay particles in soils causes shifts in clay absorption from 2,200 nm towards longer wavelengths, and therefore influencing the features at 2,400–2,500 nm [54]. On the other hand, as stated in [43], when K fertilizer is applied to soils with low clay content, K ions do not interact strongly with the soil matrix. As a result, a localized increase in K concentration in the soil solution can be observed. The influence of texture on spectral absorption of K can be seen in Figure 4(a–c). Values for absorption feature parameters: center of absorption, depth and width, are dependent on soil textural type. The figure shows how higher percentage of each textural component-clay (Clay loam, Loam, Heavy clay), sand (Fine sand, Coarse sand) or silt (Silty loam), influences the results. Therefore, the influence of soil texture prevents the computation of a universal statistical model for all textural classes.

Both experiments indicate that quantification of K depends on the amount of fertilizer added to the soil samples, as well as the soil sand-silt-clay content. In order to serve as particle tracer, the initial amount of K present in the soil should be established by collection of spectra prior to fertilizer application. This serves as a baseline and it should be accounted for when change in concentration occurs due to removal of fertilizer by erosion agents. Furthermore, particle tracing using K fertilizer is limited by K uptake by plants, and therefore cannot be performed after vegetation cover has emerged. Nevertheless, K can be quantified, through spectral response when more than 1.73 g/10 mL of K fertilizer is present in the soil. As shown in Figure 3(a–c) and Figure 5(a,c,e), the degree of change above this value becomes more constant for all textural types, while the fluctuation in parameter values for lower concentrations can be explained by noise in the spectra. In relation to common agricultural practices, this amount is the minimum quantity of needed fertilizer recommended for crop production. Higher amounts of available K in soils have not been found to cause contamination or have a negative effect on agricultural practices. This laboratory experiment to detect and quantify K concentration in soils using spectral response, suggests that the technique can be applied in field and possibly on airborne hyperspectral imagery in order to achieve rapid and extensive spatial coverage over large areas affected by soil erosion.

## 5. Conclusions

This study presents a new approach to soil particle tracing using infrared spectroscopy, allowing a rapid monitoring of soil particle movement, towards monitoring soil erosion. The aim was to find a chemical element that can replace radioactive ^137^Cs, while allowing fast and relatively inexpensive way of obtaining spatial data. Potassium is known to have the same physical and chemical behaviour in soils differing only in reactivity. Similarly to studies on ^137^Cs, near-even distribution of the element can be assumed based on the annual application of fertilizer over soil erosion affected fields. Limitations in K quantification are an unknown initial concentration of available and unavailable K, as well as the effect of uptake by plants. If fertilizer is present in concentrations higher than 1.73 g/10 mL, our technique can be applied. Best results are achieved when the method is used on soils high in sand and silt content. The defined absorption feature with a peak center positioned between 2,450 and 2,470 nm, in the infrared spectrum associated with potassium, makes it possible to detect and quantify using spectroscopy. Spectra should be acquired before and after the application of the fertilizer, to establish reference concentration.

## Figures and Tables

**Table 1.** Description of sampled soils in the Netherlands.

**Map Unit**	**Soil Type**	**Description and Properties [44]**
Rn95C	Fluvaquent-Typic	Originate from Clayey marine and fluvial sediments. Typically sandy loam or fine sand texture.
Rd10A	Fluvisol-Calcaric	Originate from predominantly recent fluvial and marine deposits. Calcaric material between 20 and 50 cm from the surface
Rn44C	Fluvisol-Eutric	Originate from predominantly recent fluvial and marine deposits. Having a base saturation of 50% or more in the major pat between 20 and 100 cm from the soil surface.
Zd21	Arenosols-Albic	Originate from unconsolidated, finely grained material. Relatively young, sandy soils with no profile development
Hd30	Podzol-Humic	Originate from weathering materials of siliceous rock. Spodic illuviation horizon under a subsurface horizon that has the appearance of ash and is covered by an organic layer
Bld6	Luvisol-Orthic	Originate from unconsolidated material. Pedogenetic clay differentiation between topsoil and subsoil. Prone to erosion due to high silt content.
